# Stimulating growth and xylindein production of *Chlorociboria aeruginascens *in agar-based systems

**DOI:** 10.1186/2191-0855-2-15

**Published:** 2012-03-12

**Authors:** Sara C Robinson, Daniela Tudor, Hilary Snider, Paul A Cooper

**Affiliations:** 1 University of Toronto Faculty of Forestry, 33 Willcocks St., Toronto, ON M5S 3B3, Canada; 2 Department of Human Biology, University of Toronto, 300 Huron St., Toronto, ON M5S 3 J6, Canada; 3 School of Forest Resources and Environmental Science, UJ Noblet Building, Michigan Technological University, 1400 Townsend Dr., Houghton, MI 49931, USA

**Keywords:** *Chlorociboria*, Green Pigment, Spalting, Xylindein

## Abstract

Four isolates of *Chlorociboria aeruginascens *were tested for possible stimulatory effects when grown on malt agar media containing wood additives. The addition of any of the four types of test wood (*Acer saccharum*, *Populus tremuloides*, spalted *P. tremuloides*, and *Ailanthus altissima*), stimulated colony growth and xylindein production in *C. aeruginascens*. Addition of any amount of wood produced more growth than no wood additions, while ground wood produced more growth than chopped wood. Of the wood types tested, *A. saccharum *wood stimulated all four isolates, while spalted *Populus tremuloides *stimulated three of the four isolates. High glucose and sucrose amounts may be partially responsible for the greater stimulatory affect of some woods over others. The development of this simple and reliable method for growth and pigment stimulation of *C. aeruginascens *in laboratory conditions will allow for further development of this fungus for decorative and commercial use.

## Introduction

The *Chlorociboria *genus is widely distributed throughout the world; *Chlorociboria aeruginascens *(Nyl.) Kanouse and *Chlorociboria aeruginosa *(Oeder) Seaver are native to North America (Ramamurthi et al. 1957). Xylindein, the penetrating blue-green pigment produced by *C. aeruginascens*, (Mizuki et al. 2003), can be readily found in forests on decaying wood, especially *Populus *sp. (Blanchette et al. 1992) and *Quercus *sp. (Dennis 1956).

Particular interest has been devoted to xylindein due to its vibrant color. The composition of this pigment has been well studied (Saikawa et al. 2000), and some research has looked at possible uses outside of the decorative market (Kunimitsu et al. 2005). Traditionally, wood stained by *Chlorociboria *sp. has been used for Intarsia inlay and other artistic media (Blanchette et al. 1992; Otterstedt 2001). Xylindein is of particular interest within the field of value-added wood products, as spalted wood can be sold at a price premium (Donovan et al. 2003), increasing the revenue of forested lands and low-value timber.

Although numerous fungus species have been researched for decorative wood staining (Robinson SC: Developing fungal pigments for 'painting' vascular plants. Appl Microbiol Biotechnol 2012), development of *Chlorociboria *sp. staining under controlled conditions has encountered several large hurdles. The native North American *Chlorociboria *sp. appear to preferentially grow on heavily decayed wood (Johnston & Park 2005), and grow very slowly both on prepared laboratory media, and in mono and dual culture jar systems (Robinson & Laks 2010a). *Chlorociboria *sp. also appears to preferentially stain some wood species over others, with *Populus *sp. and *Acer saccharum *Marsh. showing significantly more xylindein staining than *Betula *sp. or *Tilia americana *L. (Robinson & Laks 2010a).

When inducing fungal pigmentation in wood under controlled conditions, the rate of culture growth in the initial media plates is often the limiting factor. If fungi are very slow to grow and cannot completely colonize a plate, less inoculum is available for large scale lumber testing. A North American *Chlorociboria *sp. was shown to have a maximum growth diameter of 18 mm in four weeks, with neither pigment nor mycelium completely covering the plate during the entire six-month trial period (Robinson & Laks 2010a). This represents a very large problem for large-scale pigment testing, as large amounts of inoculum, whether in liquid, solid, or wood-dowel form, are necessary for inoculating logs and lumber. Fungi that cannot colonize an entire Petri plate, even under ideal growing conditions with unlimited incubation time, are not economically viable options for induced pigmentation in value-added wood.

An additional problem encountered with laboratory strains of *Chlorociboria *sp. is the tendency of some strains to stop producing xylindein in culture (Fenwick 1993), although the cause of this failure is unknown. Strains which stop producing xylindein often continue to produce only white mycelium, even after being replated onto fresh media or inoculated onto wood. If cultures cannot be guaranteed to routinely produce xylindein, this represents another hurdle in the production of *Chlorociboria *sp. stained wood for commercial purposes.

Noting the apparent preference of North American *Chlorociboria *sp. for particular wood species, this research investigated whether wood additives to malt agar plates could significantly increase the growth rate of *Chlorociboria aeruginascens*, including stimulation of xylindein production. This research also investigates conditions under which the non-xylindein producing strains might return to xylindein production.

## Materials and methods

*Chlorociboria aeruginascens *SR003, originally isolated from a decaying *Populus *sp. log in Keweenaw County, MI was grown on plates containing varying amounts and sizes of ground wood mixed into agar media. Following inoculation, the diameter of each colony was recorded.

### Preliminary testing: suspension squares versus ground wood

Several sizes and dispersion methods were utilized when making the wood-media plates. Aspen (*Populus deltoides *Bartr.), sugar maple (*Acer saccharum *Marsh.), tree of heaven (*Ailanthus altissima *(Mill.) Swingle), and spalted aspen (previously spalted with *Scytalidium cuboideum *(Sacc. & Ellis)) were chopped into roughly 5 mm × 5 mm × 2 mm thick squares. These squares were suspended in 2% malt extract agar media (100 mm × 15 mm Fisher brand polystyrene petri dishes) in either a high concentration (15-20 squares) or a low concentration (five to seven squares). Suspension was achieved by first pouring the hot media into the Petri plates, allowing the media to cool for approximately ten minutes in a laminar flow hood, then using tweezers to drop the sterilized wood squares into the media plates.

The same wood species were also ground using a Wiley mill, using both the 20 and 30 mesh screens (generating particles roughly 0.9 mm^2 ^and 0.6 mm^2^, respectively). The wood was stored in glass jars with screw caps and was autoclaved just prior to being mixed with 2% malt agar media. Two different types of wood dust plates were made: plates with a high concentration of wood and plates with a low concentration of wood. Plates containing a high amount of wood were made by adding 7.5 ml of ground wood into each Petri plate, after which the hot malt agar was poured on top and then agitated by hand using a sterilized spatula for 10 seconds. Plates containing a low amount of wood were made by mixing 2.5 ml of ground wood directly into the pre-autoclaved media (with each batch of media containing 2.5 g of malt and 1.875 g of agar and 125 mL of water), autoclaving for 15 minutes, then pouring the sterilized media into the Petri plates. Plates containing only the agar media with no wood added acted as controls. Plates were left to cool in a laminar flow hood for 24 hours prior to inoculation.

The prepared plates were inoculated with *C. aeruginascens *using a four-point inoculation. In the first round of testing, only colonies that were producing xylindein were inoculated onto the test plates. These plates were evaluated after two weeks.

### Preliminary testing: high amount versus low amount of ground wood, 20 and 30 mesh

In the second round of testing, the square piece suspension was dropped from testing, and only ground wood plates were tested. In this second round of testing, two points on each plate were inoculated from the *C. aeruginascens *isolate that produced xylindein, while the other remaining two points were taken from copies which had stopped producing the pigment. The plates were maintained in the dark at room temperature (21 C). After five days of initial growth, the widest diameter of each colony was measured using a caliper. This size was measured as the base size of the colony. Each colony was then re-measured every three to four days, and the new width of the colony was recorded. Recording was completed when inoculation points on the same media plate touched (22 days). Six replicate plates were made per wood species, per mesh size, per wood amount for all preliminary tests.

### Preliminary testing: white colonies to green

The size of white colonies was also measured, as was any green pigment produced by the white colonies during the incubation cycle. Final colony color was recorded after three weeks. Colonies were counted as green if the green pigment (assumed to be xylindein) appeared anywhere within the colony.

### Secondary testing: additional isolates

Based upon the results from the primary testing, aspen, spalted aspen, sugar maple, and tree of heaven were ground on a Wiley Mill using both a 20 and 30 mesh screen. For the secondary testing, the sterilized wood was poured directly into an empty Petri plate until the plate was half-filled with wood. Autoclaved 2% malt agar was then poured onto the wood so that the agar just covered the bottom of the Petri plate.

Three additional isolates of *C. aeruginascens *(DT8315, isolated from a decaying *Populus *log in Ontario, Canada; UAMH7614, isolated in Lake District, UK; UAMH 7615, isolated in Lake District, UK) were tested, including two that had been kept previously in refrigerated storage and were not producing xylindein on 2% malt agar plates (UAMH 7614, UAMH 7615). A two-point inoculation was used, and the same recording system as mentioned above was utilized, and data collection continued until colonies began to touch at 26 days. Three replicate plates were used per wood species per mesh size per isolate. A simple sugar analysis of test wood was conducted following the methods presented in Robinson et al. (Robinson et al. 2011), with three tested replicates per wood species. Final green colony diameters for all plates were recorded again after four months.

### Data analysis

After two weeks, colony diameter in square section suspension was compared against colony diameter on 20 and 30 ground wood plates using a 1-way ANOVA with size (square, 20 mesh, 30 mesh, no wood) as the independent variables. Follow-up testing on mesh-only plates was analyzed using a 4-way ANOVA, with wood species, wood amount (high or low), mesh size (20 or 30), and time as independent variables, and the diameter of the colony as the dependent variable. Tukey's HSD was then used to discern the location of the differences.

Data analysis on the white colonies was done using a 3-way ANOVA, with wood species, wood amount (high or low), and mesh (20 or 30) as the independent variables. Time was not used as a variable, instead readings were based upon final colony color at 45 days incubation.

For secondary testing, strains were compared using a 1-way ANOVA, followed by Tukey's HSD. Both UAMH strains were then combined into a single strain and run in a 4-way ANOVA, with strain, wood species, mesh size, and time as independent variables and colony diameter as the dependent variable. This test was followed by Tukey's HSD.

## Results

### Suspension squares versus ground wood

Square section suspension plates did not differ significantly from the controls in terms of colony diameter at the two week evaluation point, while both the 20 mesh and 30 mesh were significantly different from the control (P < 0.0001), but not from each other.

### High amount versus low amount of ground wood, 20 and 30 mesh

**Figure 1:**
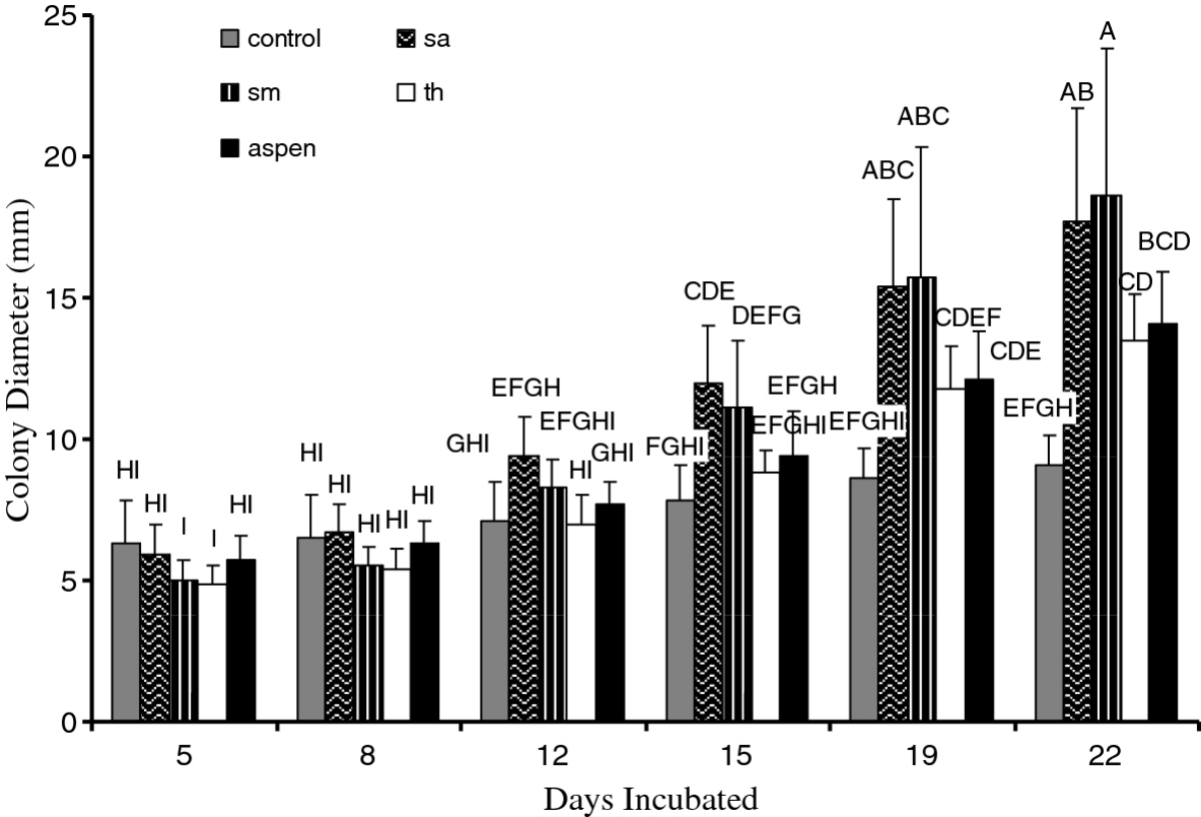
**Colony diameter of *Chlorociboria *isolates (mm) on different wood species across the testing period (22 days, second round, preliminary testing)**. Different letters represent significant differences at alpha = 0.05. sm = sugar maple, sa = spalted aspen. n = 12 replicates per wood species per week.

Within the follow-up 4-way ANOVA, mesh size was insignificant (P = 0.08). For the green colonies, wood species, time, and wood amount were highly significant (P < 0.0001), as were their interactions. Within separate 1-way ANOVAs, spalted aspen (11.2 ± 4.9 mm) and sugar maple (10.7 ± 5.9 mm) plates had significantly more xylindein than the controls (7.5 ± 1.5 mm), while aspen (9.2 ± 3.3 mm) and tree of heaven (8.6 ± 3.4 mm) did not differ significantly from the controls (P = 0.0003). Plates with the high amount of wood did not have significantly larger green colonies diameters (10.8 ± 4.7 mm) than those with the low amount (9.1 ± 4.6 mm) (P = 0.0005), although both had significantly larger diameters than the controls (7.5 ± 1.5 mm). The interaction of wood species and time shows that compared to the control plates at the final measurement period (22 days), plates containing sugar maple (at 22 days and 19 days), spalted aspen (at 22 days and 19 days), aspen at 22 days, or tree of heaven at 22 days all contained significantly larger colonies (P < 0.0001) than any other time and wood species, including the controls (Figure 1).

### White colonies to green

**Figure 2:**
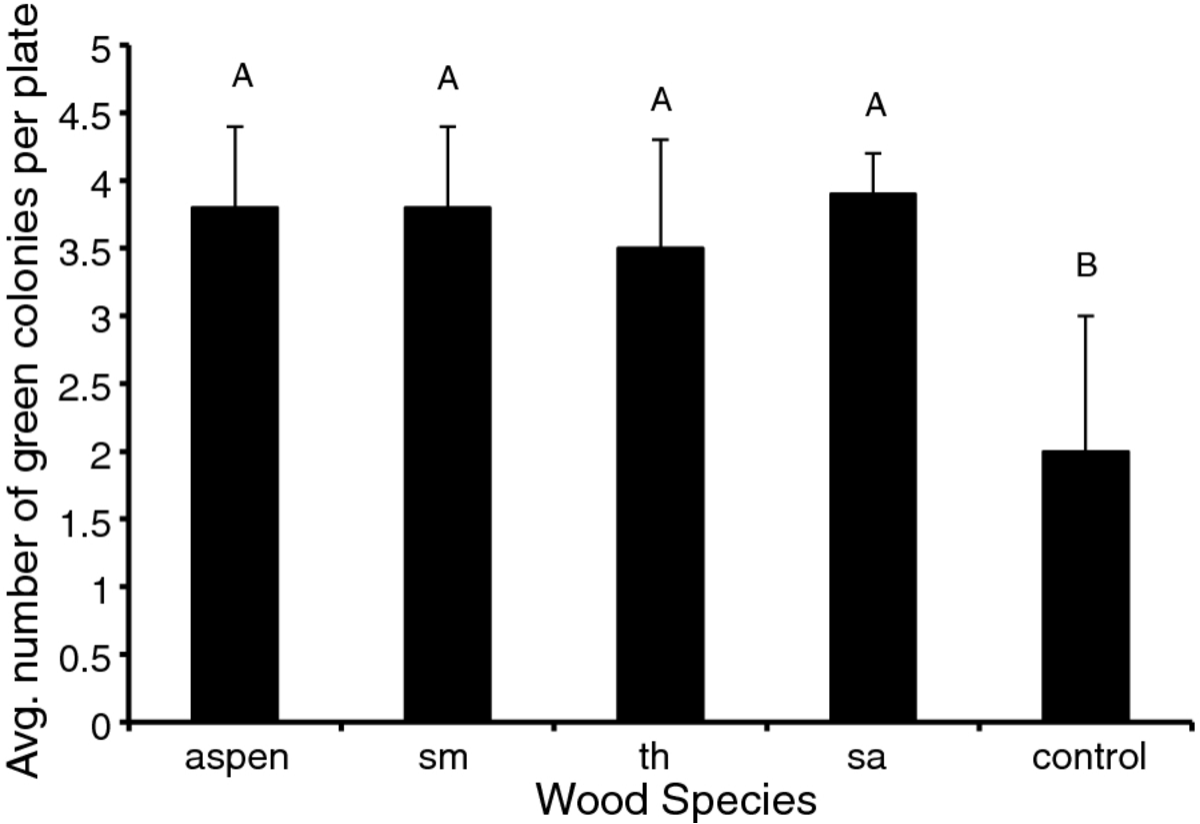
**Average number of green colonies on malt agar plates with various wood species additives**. Different letters represent significant differences at alpha = 0.05. n = 12 replicates per wood species. Error bars represent one standard deviation. Maximum number of colonies = 4. sm = sugar maple, th = tree of heaven, sa = spalted aspen.

For the white colonies, the effect of the amount of wood in the plates (high or low) was insignificant, as was mesh size or wood species. All wood species significantly increased the number of green colonies as compared to the control (P = 0.0004) (Figure 2).

### Additional isolates

Secondary testing found a difference in colony diameter by strain, with the wild strain DT8315 having larger colonies (14.4 ± 6.8 mm) than either UAMH strain (7.1 ± 4.4 mm) (P < 0.0001), which were not significantly different from each other. Within the 4-way ANOVA, mesh size was not statistically significant. Fungus strain, wood species, and time were all highly significant (P < 0.0001), as were their interactions. Sugar maple (11.8 ± 7.1 mm), spalted aspen (10.7 ± 8.0 mm), and unspalted aspen (9.5 ± 5.5 mm) plates had significantly larger colonies compared to the control (7.6 ± 2.9 mm) regardless of time or strain. Colony size increased significantly with time, although there was no significant increase in colony size between days 19 and 22, days 12 and 15, and days 8 and 12.

**Figure 3:**
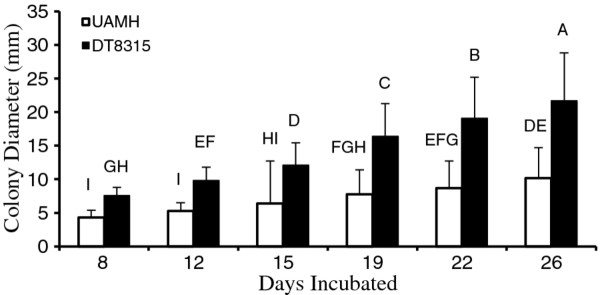
**The interaction of fungus strain and time in secondary testing (26 days)**. Different letters represent significant differences at alpha = 0.05. Error bars represent one standard deviation.

The wild strain *Chlorociboria *DT8315 consistently outperformed the two UAMH strains. For the wild strain, all wood treatments resulted in significantly larger green colonies than the control (9.6 ± 2.6 mm), with sugar maple (17.6 ± 7.7 mm) and spalted aspen (17.0 ± 7.0 mm) producing the largest (P < 0.0001). With the UAMH strains, the controls (6.6 ± 2.6 mm) did not differ from tree of heaven, spalted aspen, or aspen; only sugar maple (8.9 ± 4.6 mm) caused a significant increase in colony diameter over the control. The interaction of strain and time also showed the wild strain generally producing larger colonies than the UAMH strains, although this interaction showed areas of overlap of similar size colony production based on time (Figure 3). The three way interaction of wood × strain × time showed that within the wild strain, sugar maple (26 days), spalted aspen (26 days), and sugar maple (22 days) produced significantly larger colonies than any other pairing at any other time, including the controls (P < 0.0001).

Simple sugar content of the tested wood species can be found in Table 1. Glucose was present in all wood species, and most abundant in sugar maple. With the exception of arabinose, simple sugar content was higher in spalted aspen than in unspalted aspen.

**Table 1:** Simple sugar content (micrograms per gram) in each tested wood species

	arabinose	galactose	glucose	xylose	mannose	sucrose
**sugar maple**	not detected	not detected	1231	not detected	not detected	not detected

**aspen**	13.4	0.6	1.4	3.5	2.5	0.98

**spalted aspen**	not detected	3.3	10.1	15.8	12.6	12.1

**tree of heaven**	29.2	110	133.5	14.2	not detected	1.9

## Discussion

The addition of wood particles to agar media is used occasionally for mycological work, although its addition to growth media can affect fungi in very different ways. The addition of wood to agar induces the production of perithecium and ascospores of *Ceratocystis pilifera *(Fr.) (Zimmerman et al. 1995) and increases the growth rate of certain blue staining fungi (Shrimpton & Whitney 1968). However, wood based agar is also occasionally utilized as a way to limit growth on media (Lonergan et al. 1993). It is apparent from this study that the addition of any wood to media plates inoculated with *C. aeruginascens *does not retard growth or xylindein production, and in many cases appears to stimulate both.

In tests comparing lower amounts of wood in plates to higher amounts, plates with more wood routinely had more fungal pigment and larger colonies. It is possible that *C. aeruginascens *requires a nutrient not found in malt agar media; however continued stimulation with increasing wood amounts suggests an additional unknown element at play. A similar phenomenon was found by French and Manion (French & Manion 1976), who found the growth rate of *Hypoxylon mammatum *colonies increased with increasing concentrations of ground wood in their media. However, in their study, wood tissue was utilized as the only nutrient base for the agar.

**Figure 4:**
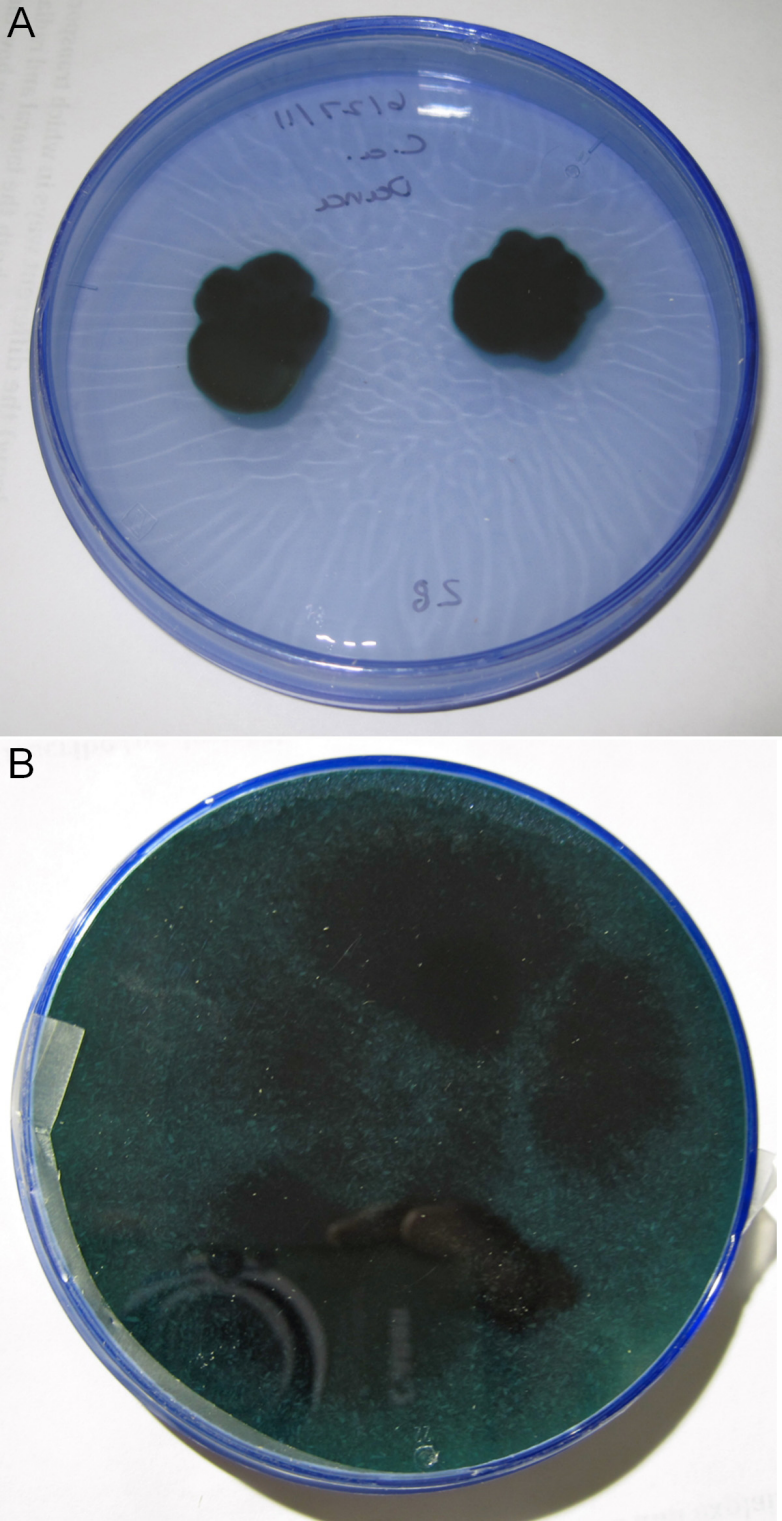
**Growth and xylindein production on malt agar media at four months**. (a) Media without wood additives, strain UAMH 7615, (b) media with high amounts of sugar maple, 30 mesh, strain UAMH 7615.

When compared to the maximum colony diameter reported in Robinson and Laks (Robinson & Laks 2010a), it appears that the addition of any wood additive to media increases both colony diameter and xylindein production (Table 2 Figure 4). Most plates with wood additives showed complete colonization (100 mm max possible diameter per colony with a four point inoculation on 100 mm Petri plate) at the four month evaluation point which, although still a slow growth rate, is a strong increase from the maximum 18 mm colony diameter (on 100 mm Petri plate) reached by the previously reported isolate, as well as substantially larger than the maximum control diameters for each tested isolate. Although some variability existed among the four strains tested, sugar maple routinely significantly increased colony diameter and promoted xylindein production in non-pigmented sections. The effects of the other wood species varied by *C. aeruginascens *isolate, although spalted aspen also stimulated growth significantly in isolates SR003 and DT8315.

**Table 2:** Comparison of five *Chlorociboria *isolates and their maximum colony diameter growth.

Isolate	wood species	max diameter (mm)	# months
Previous isolate	none	18	6
SR003	none	17.8	4
	sugar maple	Maximum diameter	4
	spalted aspen	Maximum diameter	4
	aspen	Maximum diameter	4
	tree of heaven	Maximum diameter	4
DT8315	none	18.4	4
	sugar maple	Maximum diameter	4
	spalted aspen	Maximum diameter	4
	aspen	Maximum diameter	4
	tree of heaven	Maximum diameter	4
UAMH7614	none	20.54	4
	sugar maple	Maximum diameter	4
	spalted aspen	Maximum diameter	4
	aspen	51.07	4
	tree of heaven	Maximum diameter	4
UAMH7615	none	27.6	4
	sugar maple	Maximum diameter	4
	spalted aspen	Maximum diameter	4
	aspen	54.1	4
	tree of heaven	Maximum diameter	4

Complete coverage indicates that both the xylindein and colony have covered the entire plate. Plate dimensions = 100 mm × 15 mm, maximum possible diameter = 100 mm

The reasons for the apparent wood preference in *Chlorociboria *sp. is unknown; however a recent study of several other pigment-producing fungi hypothesized that higher sucrose levels in some wood species may be a contributing factor (Robinson et al. 2011). Simple sugar analysis of the tested wood species in this experiment found a higher level of sucrose and mannose in spalted aspen over the other wood species, although sugar maple did not contain a detectable amount of either. In contrast, the glucose level of sugar maple was much higher than the other wood species, including spalted aspen. It is possible that a combination of the two sugars, sucrose and glucose, plays a role in stimulation of fungal growth and pigment production. However, the simple sugar analysis does confirm the results of Robinson et al. (Robinson et al. 2011) and Anagnost et al. (Anagnost et al. 1994), both of which noted an increase in galactose in wood colonized by *S. cuboideum*.

There are several possibilities to explain the variability among the isolates. Both isolate SR003 and isolate DT8315 were isolated from North America, and neither has ever been stored outside of room temperature on malt agar media. Both UAMH isolates are part of the large University of Alberta culture collection, and originated in the UK. As both UAMH isolates performed similarly, as did both North American isolates, it is possible that the strains differ based on region of origin. Culture age may also play a role. The North American strains were isolated within the past five years, whereas the UAMH strains, originally isolated by Fenwick, appear to be part of his work published in 1993. Culture age is known to affect the pigment output of some fungi (Robinson & Laks 2010b), with increasing culture age related to a decrease in pigment output.

It is interesting to note that all strains of *C. aeruginascens *produced a yellow pigment when inoculated onto sugar maple media. This pigment was visible after five days of incubation, spreading throughout the entire plate by 10 days. This yellow pigment has been reported before, and may be a xylindein quinol, which is occasionally also produced by *Chlorociboria *species in addition to xylindein (Edwards & Kale 1965).

The addition of sugar maple as an additive to agar-based growth media for *C. aeruginascens *may be the key to this fungus becoming an economically viable option for induced wood pigmentation. Although the colony growth rate of *C. aeruginascens *is still slower than most routine spalting fungi (*S. cuboideum*, *Trametes versicolor *(L.) Lloyd, *Xylaria polymorpha *(Pers.) Grev.), the ability to achieve full plate colonization and pigmentation of albino colonies will allow log and lumber scale use of *C. aeruginascens *as a spalting fungus to develop.

## Competing interests

The authors declare that they have no competing interests.
